# Early Childhood Caries, Masticatory Function, Child Early Cognitive, and Psychomotor Development: A Narrative Review

**DOI:** 10.1055/s-0043-1774326

**Published:** 2023-12-04

**Authors:** Taufan Bramantoro, Fredy Mardiyantoro, Wahyuning Ratih Irmalia, Risma Aprinda Kristanti, Alexander Patera Nugraha, Tengku Eleena Binti Tengku Ahmad Noor, Asra Al Fauzi, Udijanto Tedjosasongko

**Affiliations:** 1 Department of Dental Public Health, Faculty of Dental Medicine Universitas Airlangga, Surabaya, East Java, Indonesia; 2 Department of Oral and Maxillofacial Surgery, Faculty of Dentistry, Brawijaya University, Malang, East Java, Indonesia; 3 Dental Public Health & Primary Health Care Research Group, Faculty of Dental Medicine, Universitas Airlangga, Surabaya, Indonesia; 4 Department of Biomedical Science, Medical Study Program, Faculty of Medicine and Health Science, UIN Maulana Malik Ibrahim Malang, Malang, East Java, Indonesia; 5 Department of Orthodontic, Faculty of Dental Medicine Universitas Airlangga, Surabaya, East Java, Indonesia; 6 Department of Periodontology, Membership of Faculty of Dental Surgery, Royal Collage of Surgeon, Edinburgh University, Edinburgh, United Kingdom; 7 Department of Neurosurgery, Faculty of Medicine Universitas Airlangga, Surabaya, East Java, Indonesia; 8 Department of Pediatric Dentistry, Faculty of Dental Medicine Universitas Airlangga, Surabaya, East Java, Indonesia

**Keywords:** caries, medicine, masticatory dysfunction, cognitive development, psychomotor development

## Abstract

Dental caries is known as a global public health issue that has been affecting general health apart from its painful nature. Hence, it is undeniable that caries affecting young children or known as early childhood caries, also have an effect on children's general health. One of the interesting findings about caries is that it can also affect child growth and development, specifically on their cognitive and psychomotor ability. Untreated caries are linked to cognitive development through both neural and vascular pathways, with masticatory function as the key. Meanwhile, its effect on psychomotor development might be related to nutritional intake, which might slightly decline on those with caries. This review is aimed to describe the current findings of caries effect on early child development, from masticatory disturbance to further impacts on cognitive and psychomotor development. The overall conclusion of this review is that untreated severe caries in children are potentially associated negatively with their growth and development.

## Introduction

Oral diseases pose as one of the main worldwide public health issue. Early childhood caries (ECC) is a significant oral health problem, which is characterized by the existence of one or more decayed (both cavitated and noncavitated lesions), missing, or filled teeth in the primary dentition of children aged 71 months or younger. 1 It is considered as a chronic infection that most frequently affects children. 2

Considering its unidirectional and bidirectional effects, oral health is considered one of the essential elements of well-being and general health. 3 4 One of the most interesting factors is the correlation between mastication impairment, either due to untreated caries or early tooth loss, and cognitive function. 5 6 7 To date, several studies had discovered its correlation with cognitive function, specifically in elderly. These studies found that those with more tooth loss are more susceptible to dementia. 8 9 Fortunately, research on animals found that this effect is reversible with proper treatment and care. 10

Child development is categorized into four domains, namely physical development, cognitive development, linguistic development, and socioemotional development. Physical development includes growth rate, physical fitness, gross and fine motor skills development, and self-care ability. Cognitive development consists of intellectual processes of analysis, problem-solving, early mathematical ability, and memory. Linguistic development consists of babbling, gestures, and pointing in infancy. Socioemotional development denotes the child's relationship with caregivers and how they build trust to fulfill their needs. 11

Considering the high number of children suffering from ECC in developing and industrialized countries, 12 especially the socially disadvantaged population, a further understanding of the effects of ECC on children's general health is necessary. 3 One of the significant effects of caries is deterioration in the ability to chew, 13 which further affects their nutritional intake, pain, and quality of life. 4 In addition, previous studies have found other negative effects of ECC on children's growth, such as psychomotor function and development, 11 14 15 16 17 cognition, 18 speech development, 3 11 19 as well as their well-being in adulthood. 20 Therefore, this article aims to provide a summary of the present literature about ECC and its effects on masticatory function, cognition, and other aspects of child development.

## Early Childhood Caries

Dental caries is widely known as a preventable disease. However, it immensely affects disadvantaged groups from the beginning of life through to the elderly. Likewise, the increase in oral health disparities has caused caries to be declared a silent epidemic. 21 ECC is dental health problem that is commonly found in children, with the incidence reaching 1.76 billion, regardless of their socioeconomic status. 2 Aside from ECC, the following names are also used reciprocally: nursing caries, rampant caries, early childhood tooth decay, baby bottle-fed tooth decay, comforter caries, and maxillary anterior caries. 12 The problem of children suffered from ECC is not as simple as due to the caregiver do not maintain their oral health. In addition to dental health disparities, the families may face certain conditions forcing them to pay less attention to their children's dental care. 19

ECC that affects children or toddlers is quite distinctive. 12 It is characterized with existence of one or more decayed (both cavitated and noncavitated lesions), missing, or filled teeth in the primary dentition of children aged 71 months and younger. 1 Any signs of caries on the smooth surface of the teeth in children under the age of 3 are indicators of severe early childhood caries (SECC). 22 In addition, any missing teeth due to caries, or restored primary teeth in the maxillary anterior region, or decay, missing, filling (dmf) scores more than or equal to 4 at the age of 3, more than or equal to 5 at the age of 4, or more than or equal to 6 at the age of 5 years are also considered as SECC. 12 The initiation stage of ECC is indicated by a dull white spot of demineralized enamel that rapidly advances into noticeable decay along the gingival margin. The first affected teeth are usually the primary maxillary incisors, all at once. The lesions can be found on either lingual or labial surfaces or, sometimes, both. 12

Early childhood life denotes one of stage of life that is vulnerable to health problems. Aside from their independence, it is also difficult for young children to convey what they need. As a result, their health and well-being heavily rely on the beliefs and health practices of their primary caregivers. 22 One of the triggering factors for the occurrence of ECC is the transmission of the main bacteria that causes dental caries, namely *Streptococcus mutans* ( *S. mutans* ). Children acquire the *S. mutans* from their closest person or caretakers. 23 Research shows that the younger a child acquires *S. mutans* , the higher the risk of the child experiencing caries. 24

Studies have shown that ECC somewhat impacts on children's quality of life, such as pain arises from the untreated caries, eating difficulties, sleep problems, developmental issues, school absences, and social self-consciousness. 21 The occurrence of dental caries in children is inseparable from the occurrence of dysbiosis in the oral microbiome, which can also cause dysbiosis in the gastrointestinal microbiome. 25 This, in turn, can lead to various health problems in children. Apart from the child itself, ECC also affects their family through financial aspect due to dental treatment expenses and potential time off work to look after the unhealthy children. 3

Previous studies have revealed that high carious activity is associated with various aspects of general health, such as early tooth decay, orofacial pain, 2 26 27 reduced dietary intake, 27 28 weight loss, sleep disturbances, hindered school, and learning activities, ultimately resulting in a poorer quality of life. 29 ECC can also cause masticatory dysfunction, 30 affecting intellectual development and other developmental milestones. 14 18 31 32

## Early Childhood Caries Experience and Masticatory Function

Untreated caries directly affects the mastication unit. Loss of occlusal contact or tooth extraction may disrupt the permanent teeth alignment, increasing the risk for orthognathic problems later in life. Mal-aligned teeth and masticatory problems may subsequently make children more susceptible to periodontal disease. 22 Studies have found that cavitated teeth might affect children's ability to chew food, 26 33 34 especially in children with a greater number of missing teeth. 20 26 As a result, they prefer to eat soft diets. 35 However, a study has revealed that the negative effects of caries on mastication are reversible. 10 Dental restoration treatment for caries is able to restore oral function by increasing their bite force value. 36 37 Meanwhile, another study suggested that despite the difficulty in chewing due to extensive tooth extraction, children are able to adapt, and achieve optimal masticatory function. 35

The mechanism behind the changes in oral function due to caries is that extensive carious lesions disrupt the tooth structure, leading to open occlusal or proximal contacts. This results in reduced bite force due to a smaller area for grinding food. Consequently, this might lead to selective food texture, prolonged chewing time, and one-sided eating. Likewise, dental pain may also arise during eating, causing sensitivity and difficulty in chewing. 13 38 39

## Mastication Impairment and Cognitive Function

Children with untreated caries are prone to mastication impairment. There are studies suggesting the detrimental effects of mastication impairment on hippocampus-related cognitive function. These effects can occur through both the neural pathways 40 41 and the decrease in blood flow. 42 Mastication denotes cyclic, repetitive motions of the jaw, regulated by the feedback signals from periodontal mechanoreceptors. These receptors are widely recognized as the main source of signals containing information about the initial contact between teeth and food. The central nervous system processes this information to control masticatory muscle and jaw movements. 43 While the main purpose of these signals is to control masticatory movements, these somatosensory inputs also influence the hippocampus through synaptic projections from the thalamus and cerebral cortex. 44 Stimuli from masticatory movements play an important role in maintaining social, mental, and physical health. The ability to chew is associated with overall health through nutritional status and daily activities. The two parts of the central nervous system that regulate cognitive function are the cerebral cortex and hippocampus. 45

The mastication process generates sensory signals that are transmitted through the trigeminal sensory nerves to various brain regions, including the brainstem reticular formation, cerebellum, hypoglossal motor nuclei, and trigeminal sensory nuclei. 46 47 48 The trigeminal sensory nerves later arrive in the ventral posterior thalamic nucleus, reticular formation, and hypothalamus. The sensory signals from the ventral posterior thalamic nucleus ultimately reach the somatosensory cortex. Nerve fibers from the somatosensory cortex project their axons to the somatosensory association area, which is connected to the entorhinal cortex. The entorhinal cortex is the primary afferent source for the hippocampal dentate gyrus, allowing sensory signals from the mastication process to affect the hippocampus via the thalamus and cerebral cortex. The hippocampus plays a crucial role in regulating the release of various hormones from the hypothalamic-pituitary-adrenal axis. The hypothalamus produces corticotropin-releasing hormone, which triggers the anterior pituitary gland to secrete adrenocorticotropic hormone, causing the release of corticosterone from the adrenal cortex. 6 40 Corticosterone has quick access to the brain. The hippocampus has a large number of glucocorticoid receptors, triggering the release of stress-related hormones. 49 The hippocampus receives projections of noradrenergic, serotonergic, and dopaminergic fibers from the locus coeruleus, raphe nuclei, and ventral tegmental area, which are parts of the ascending reticular activating system. Therefore, it can be concluded that the mastication process can affect the function of the hippocampus through reticular formation, and the effects of mastication on the hippocampus can arise from several neural pathways. 41 49

The mastication process produces sensory signals to the hippocampus. 50 If the process does not occur optimally, it will cause disturbances in the morphological development of the hippocampus, such as a decrease in the number of pyramidal cells. 40 Pyramidal cells are cells in the cerebral cortex that send nerve fibers to the spinal cord and terminate efferent motor neurons that innervate skeletal muscles. 51

In addition to the neural pathways, mastication movements can affect the brain through an increase in the cortical blood flow, indicating a response from sensory signals from the masticatory system toward the brain. Furthermore, the increasing partial pressure of carbon dioxide due to feedback mechanisms from sensory and motor neurons in the cortex results in dilated capillaries. 42 50

Mastication has been associated with various cognitive functions, including episodic memory, verbal fluency, psychomotor function, and delayed word recall. These functions are regulated by specific parts of the cerebral cortex and hippocampus. 45 Observational studies using positron emission tomography and functional magnetic resonance imaging showed that masticatory muscle activity increases cortical blood flow and activates areas such as the supplementary motor area, somatosensory area, insular cortex, cerebellum, and thalamus. 48 52 53 In addition, an increase in blood supply to the hippocampus and prefrontal cortex was observed during activities following mastication. This activity is crucial for the learning and memory process. Finally, the increase in blood flow to the brain resulting from mastication is considered to have beneficial effects on a wide range of cognitive functions. 45

Epidemiological studies have shown that the number of remaining teeth, denture wear, and low bite force are associated with an increased risk of dementia. 41 Another study also showed that reduced mastication can lead to mild cognitive decline in Korean adults. 54 Other studies using animals with masticatory impairment due to tooth extraction, 55 56 occlusal bite plane, 57 58 and soft diet 59 found that these conditions altered hippocampal function in the spatial memory and learning ability. However, other studies also found that the declining function of the hippocampus can be restored by increasing chewing activity. 10 47

To date, there are studies that have found a correlation between masticatory ion impairment and specific food texture preferences in both human and animal subjects. However, to the best of our knowledge, most studies have mainly focused on the elderly, suggesting that masticatory impairment increases the risk of dementia and worsens their cognitive function. A study investigating reduced mastication in young mice found a correlation with low hippocampus activity, causing changes in the morphology of the hippocampus. 7 60 This could result in impaired function in spatial memory and learning ability 7 as well as increased vulnerability to mental disorders. 60 Although other studies have also found similar effects in post-weaned rats, 44 no such evidence in children has been found. Only a few articles mention that caries in children might affect their cognitive function and neurodevelopment. 18

**Fig. 1:**
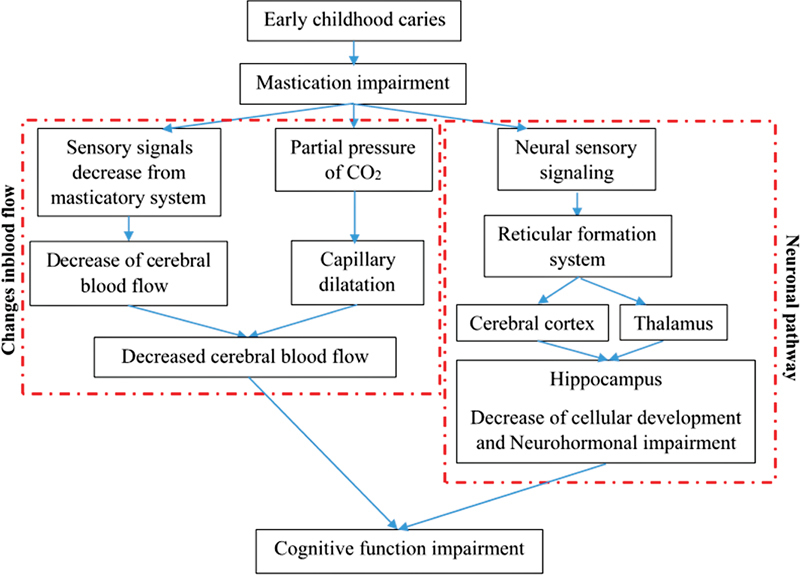
The effects of early childhood caries on cognitive function through changes in blood flow and neural pathway.

The chewing patterns of children are different from those of adults. In addition, several jaw movement parameters change with age. In early life, the sensorimotor control of jaw movements adapts to morphological changes resulting from growth. Children can perform adult-like chewing behavior as they reach the late-permanent dentition phase. Changes in the histological imaging of periodontal mechanoreceptors during human growth are currently unknown. However, research on animals shows that the density of periodontal mechanoreceptors in the primary dentition is lower compared with the permanent dentition. Therefore, it can be assumed that the periodontal mechanoreceptors provide inadequate and immature sensory inputs during primary and mixed dentition. 43 This could explain the lack of published articles investigating the effects of masticatory ion impairment, specifically due to ECC, on cognitive function and neurodevelopment in children. Further research regarding the effects of untreated caries, early primary tooth loss, mastication difficulties on children's cognitive function and neurodevelopment is required. The summary of the effects of ECC on cognitive function is presented in Fig. 1 .

## Effect of Early Childhood Caries and Psychomotor Function

The process of human growth from infancy to adulthood involves both mental and physical development. 15 While cognition refers to the acquisition of knowledge through intellectual processes, psychomotor function refers to the acquisition of behavior through neuromuscular motor activities. 61 The psychomotor function includes the combination of precise motor responses, attention, and cognitive problem-solving abilities. Psychomotor development represents children's growth in terms of cognitive, emotional, social, and motor skills from early life. 62

To date, ECC and psychomotor deficiency appear to be physiologically correlated. Although somewhat elusive, it can be assumed that high caries activity makes children prone to reduce chewing and swallowing activities, leading to inefficient digestion, which, in turn, results in a lack of nutrition required for growth. 14 Studies have found that children aged 4 to 6 years old with dmft scores more than or equal to 4 was significantly associated with psychomotor deficiency (e.g., comprehension-concept and expressive language). However, children with dmft scores more than 6 showed no delay in personal, social, and expressive language development. 11 15 Similar results were also discovered by another study, showing that more severe ECC with dmft scores of between 3 and 8 affected the children's psychomotor development, specifically in expressive language and comprehension concepts. 16 These studies found a negative correlation between caries and psychomotor function, suggesting that higher dmft scores are associated with less language development. This might potentially affect both expressive and receptive abilities in growing children. 14 16

To be able to communicate effectively, children must be able to produce meaningful sounds, which involve the coordination of respiration, voice, and articulation movements. To produce correct phonemes, coordinated movements involving the jaw, lips, tongue, soft palate, teeth, and upper airway (pharynx) are required. Maxillary incisors are associated with the articulation of l, n, d, and r sounds. 63

Despite the new finding, the mechanisms behind the potential effects of ECC and psychomotor impairment remain unclear. 14 15 16 17 Further research is necessary to determine whether the correlation between ECC and psychomotor impairment is established directly via the neurophysiological pathways or other pathways outside the nervous system. However, this correlation can be attributed to several pathways. ECC can affect children's growth, including physical, psychomotor, or psychological, due to pain from cavitated teeth or primary tooth loss, which can alter nutritional intake. This could potentially lead to nutrient loss, low body weight, sleep disturbances, and ultimately a poorer quality of life. In addition, the presence of any confounding factors (e.g., gender and age) and comodifiers (i.e., external behaviors or other environmental factors affecting psychomotor function) should be further explored to verify their potential impacts on this new finding. 14 Another possible mechanism is that severe ECC can compromise mastication forces, verbal skills, and brain metabolism. These effects, if persist for a long time, may likely alter the children's interactions with others, and further affect their social engagement or learning, consequently impacting their language expression, verbal skills, and communication. Therefore, ECC could likely trigger the related development delays over time. 14 15 16

**Fig. 2:**
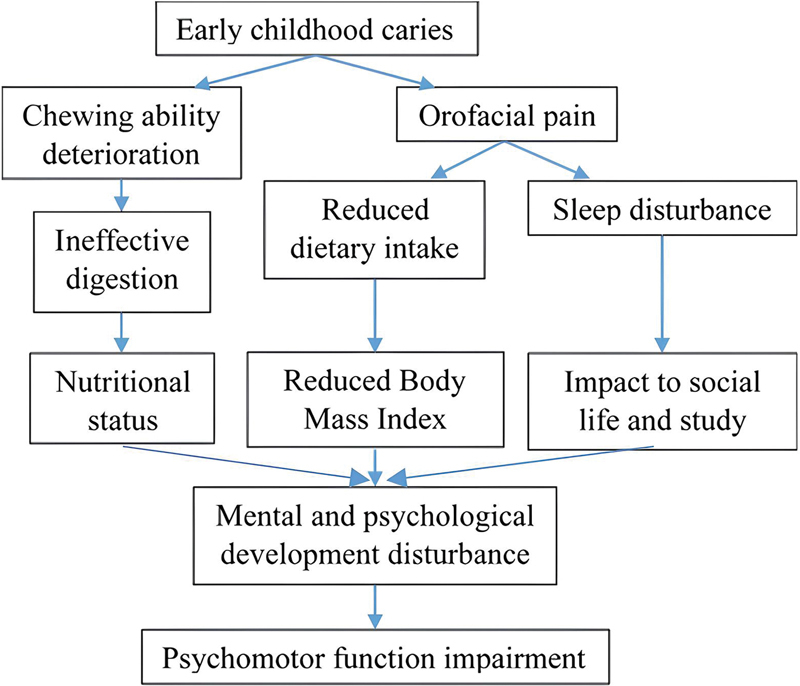
The potential effects of early childhood caries on psychomotor function.

The pathways showing the effects of ECC on psychomotor function are presented in Fig. 2 . It is expected that children with severe untreated caries are likely to experience chewing difficulties, resulting in inefficient digestion which further affects their nutritional intake. The lack of nutrition can subsequently affect their development, learning, and emotional maturity. 15

## Conclusion

In summary, this study revealed that untreated severe caries in children is negatively associated with their growth and development. The most apparent effects are masticatory impairment, nutritional intake, and verbal skills. However, the effects of untreated severe caries on cognitive function need further exploration to determine whether masticatory dysfunction affects cognitive function in young children as suggested by research on animals. The relationship between ECC and psychomotor is complicated, considering the potential interplay of causal factors, confounding variables, risks, and interference. Before arriving at a definitive conclusion, further investigation should focus on how ECC might influence sound distortion, disarticulation, or poor oral functions, such as physical malocclusion and deficient language development during maturation.
